# A mobile robotic approach to autonomous surface scanning in legal medicine

**DOI:** 10.1007/s11548-025-03507-w

**Published:** 2025-09-12

**Authors:** Sarah Grube, Sarah Latus, Martin Fischer, Vidas Raudonis, Axel Heinemann, Benjamin Ondruschka, Alexander Schlaefer

**Affiliations:** 1 Institute of Medical Technology and Intelligent Systems, Hamburg University of Technology, Hamburg, Germany; 2 Faculty of Electricity and Electronics, Kaunas University of Technology, Kaunas, Lithuania; 3 SustAInLivWork Center of Excellence, Kaunas, Lithuania; 4 Institute of Legal Medicine, University Medical Center Hamburg-Eppendorf, Hamburg, Germany

**Keywords:** Legal medicine, Point cloud stitching, Parametric study, Configuration space analysis, Patient documentation

## Abstract

****Purpose**:**

Comprehensive legal medicine documentation includes internal and external examination of the corpse. Typically, this documentation is conducted manually during conventional autopsy. Systematic digital documentation would be desirable, especially for external wound examination, which is becoming more relevant for legal medicine analysis. For this purpose, RGB surface scanning has been introduced. While manual full-surface scanning using a handheld camera is time-consuming and operator-dependent, floor or ceiling-mounted robotic systems require specialized rooms. Hence, we consider whether a mobile robotic system can be used for external documentation.

****Methods**:**

We develop a mobile robotic system that enables full-body RGB-D surface scanning. Our work includes a detailed configuration space analysis to identify the environmental parameters that must be considered for a successful surface scan. We validate our findings through an experimental study in the lab and demonstrate the systems application in legal medicine.

****Results**:**

Our configuration space analysis shows that a good trade-off between coverage and time is reached with three robot base positions, leading to a coverage of 94.96 %. Experiments validate the effectiveness of the system in accurately capturing body surface geometry with an average surface coverage of ${96.90 \pm 3.16}$ % and ${92.45 \pm 1.43}$ % for a body phantom and actual corpses, respectively.

****Conclusion**:**

This work demonstrates the potential of a mobile robotic system to automate RGB-D surface scanning in legal medicine, complementing post-mortem CT scans for inner documentation. Our results indicate that the proposed system can contribute to more efficient, autonomous legal medicine documentation, reducing the need for manual intervention.

**Supplementary Information:**

The online version contains supplementary material available at 10.1007/s11548-025-03507-w.

## Introduction

In legal medicine, documentation of corpses is a common and increasingly important task, serving multiple purposes such as documenting injuries for medico-legal investigations and providing evidence for legal proceedings. To achieve comprehensive documentation, it is essential to analyze not only the internal structures of the body, but also to accurately document the external surface of the body. By integrating surface and internal structure data, the aim is to create comprehensive legal medicine records [3, 6, 26, 29].

While post-mortem computed tomography (PMCT) and post-mortem magnetic resonance imaging (PMMRI) provide detailed insights into internal anatomy and pathology, these modalities are limited in capturing detailed surface information, such as skin color and the finer details of surface injuries. Therefore, body surface analysis is decisive in accurately documenting the external shape and color of abnormalities such as wounds.

Various system approaches have been used for documentation [2]. Conventionally, 2D imaging using digital cameras has been used. However, advances in technology have led to more sophisticated methods, particularly 3D surface scanning techniques. Photogrammetry, for example, uses multiple images taken from different angles with a handheld camera, camera rig or a special rod to create a 3D model [10, 19, 27]. Alternatively, 3D surface scanning with handheld time-of-flight cameras is used. These approaches offer mobility and flexibility, allowing the scanning system to be used in different rooms and environments, hence not limiting the system to a single fixed configuration. Despite these advances, there are trade-offs within these approaches, including cost efficiency, mobility, repeatability and automation. Cost-effective approaches typically require manual and well-trained operation with a lack of automation. Automated approaches tend to be expensive and require a fixed setup to ensure consistent positioning of the scanned body relative to the system [6, 10]. A cost-effective approach is presented in Sieberth et al. [27], using a spoke rod with multiple cameras. However, manual scanning is required.

There is growing interest in automating these procedures using robotic systems [12] to make the documentation process more time-efficient and to minimize the risk of infection. The swiss Virtopsy movement [26] focuses on providing high-resolution multi-modal 3D color documentation of corpses. As part of the Virtopsy project, the Virtobot was developed [6] for automated surface scanning using predefined robot positions. This system already shows promising results at the Institute of Forensic Medicine in Zurich. However, the high costs, a fixed large installation with specific ceiling requirements to mount the robot, the required thorough preparation of the body, and the application of multiple markers to the body for accurate data stitching make this setup less flexible. Together with the necessary manual path planning of the robot, these requirements limit the autonomy of the system.

In summary, the need for automated, time-efficient and multi-modal solutions for legal medicine documentation is growing and increasingly important. Such solutions should be low-cost and mobile with respect to different space constraints and autonomous to reduce the workload and minimize the risk of infection.

In this article, we present a mobile and autonomous surface scanning robot for comprehensive external examination and digital documentation of corpses, including for complementing PMCT scanning or pre-autopsy documentation. In our context, autonomous refers to a robot that can recognize the pose of a body, independently decide which positions are best for surface acquisition, and independently perform the scanning process. A robot base with differential drive enables flexible positioning of a lightweight 6-joint robot arm with an RGB-D camera attached for surface scanning, capturing color (RGB) and depth (D) information. The mobile base allows us to increase mobility and adaptability by extending the operating range of a robotic arm, providing a more autonomous alternative to traditional approaches. The RGB-D information is used both for autonomous control and navigation of the robotic setup in the current environment and for surface scanning.

Our experimental evaluation aims to investigate the performance of our system for comprehensive surface scanning. First, we investigate which environmental settings have to be considered in order to successfully perform a surface scan and how many different robot base positions are needed. We use a configuration space analysis, to perform a detailed parametric analysis, investigating factors such as different workspace constraints, body geometries, and camera positioning, as well as the trade-off between scanning time and surface resolution. Second, we validate our results with real-world experiments in a laboratory setting and demonstrate the application of our system in legal medicine.

**Fig. 1:**
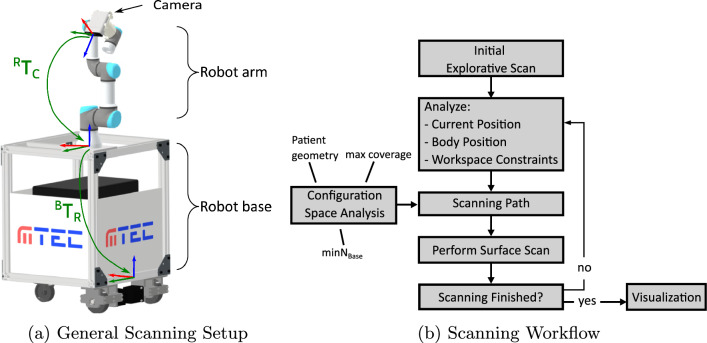
General System Setup: Schematic drawing of the mobile scanning setup (left) and the corresponding scanning workflow (right)

## Material and methods

### General setup and workflow

Our mobile robotic system depicted in Fig. 1a is designed to enhance autonomous surface scanning. The system consists of a mobile base that allows flexible positioning of a robotic arm around the body. We refer to the mobile base as robot base. The robotic arm is equipped with an RGB-D camera for surface scanning and navigation. The proposed workflow of an autonomous 3D surface scanning procedure using our system is shown in Fig. 1b and in the video (Online Resource 1).

A configuration space analysis is conducted once, based on the given robotic system parameters, to define scanning paths that ensure maximum coverage of a body geometry while minimizing the number of robot base positions. Our scanning workflow starts with an initial explorative scan to assess relevant environmental factors such as the initial orientation of the patient and robot, spatial constraints such as obstacles, couch height and body position. Subsequently, the initial explorative scan is combined with the conducted configuration space analysis to select an optimal scanning path considering the identified environmental constraints. Path planning for the mobile robot base is performed using an A* algorithm [9]. Surface scanning is performed by iteratively moving the robot base and robot arm following the predefined optimal scanning path. Once the body has been scanned, post-processing steps are performed and the surface scan is displayed to the clinician.

### System parameters and calibration

Our autonomous scanning system consists of a small, lightweight robotic arm (UR3, Universal Robots, DK) mounted on a differential drive robot base (two wheels driven by Stepperonline Servo T6M80 motors with T6-750RS controllers). An RGB-D camera (Azure Kinect, Microsoft, USA) is attached to the fifth joint of the UR3. We communicate with the differential drive base via an Arduino, with the robot base velocity limited to 0.08 m/s for safety reasons. We use the Robot Operating System (ROS 1)  [24] to control the entire system. The UR3 robotic arm is controlled using MoveIt! [4]. Through MoveIt!, we also utilize third-party libraries, including the Universal Robot Driver [28], the Kinematics and Dynamics Library [1], the Fast Collision Check Library [16], and the Open Motion Planning Library [25].

Initially, hand-eye calibration using Parks method [17] is performed to obtain the transformation $^RT_C$. Using an 8x5 tile checkerboard, we acquire 100 images for random robot arm configurations. We obtain reprojection errors of 0.039 and 0.271 px for RGB and IR imaging, respectively. For hand-eye calibration we obtain a translation error of 6.21 mm (x= 2.26 mm, y= 4.72 mm, z= 2.34 mm) and a rotational error of 0.423$^\circ$. We define the transformation $^BT_R$ between the UR3 base and the robot base based on the kinematic design.

### Configuration space analysis

We aim to systematically identify the necessary robot configurations to enable a comprehensive scanning of a specific body geometry. A configuration space analysis is performed that optimizes with respect to both the maximum coverage of the body surface and the minimum number of robot base positions used. The configuration space of the scanning robot is defined by the position of the robot base and the configuration of its arm. We assume the best visibility when the camera is orthogonal to the body surface. Consequently, for each robot base position, we determine which body surface points can potentially be seen and which configurations of the robot arm are required. Finally, the configuration space analysis provides a dictionary of all configurations and the corresponding visible points.

### Selecting the scanning path

In a subsequent step, the dictionary from the configuration space analysis is used to identify the most promising robot base and robot arm configurations for scanning the body surface. This process uses a greedy algorithm to iteratively add the robot configuration with the largest number of currently unseen surface points until no new points can be captured. Note that changing the camera field of view by moving the robot arm is faster than changing the robot base position. Therefore, we first move the robot arm to reach unseen surface points.

### Reconstruction of surface scan

A full-body surface scan is reconstructed by stitching the acquired point clouds based on the respective robot configurations. We first merge the captured point clouds for the individual robot base positions and then arrange these in space. We then refine the alignment performing a global RANSAC registration [8] based on feature matching, followed by using a point-to-point Iterative Closest Point (ICP) algorithm [20]. Finally, we perform outlier removal and cropping to the region of interest by detecting and isolating the couch and patient area based on its shape. Point cloud processing steps are implemented using functions provided by the Python Open3D library [31]. To reduce computation time, point clouds are down-sampled to a resolution of 10 mm.

### Experimental evaluation

The experimental evaluation is divided into a computational analysis of the configuration space and corresponding scanning paths and two experimental studies applying the robotic setup in both a laboratory and a clinical setting.

#### Configuration space and path evaluation

A detailed configuration space analysis is performed to investigate the influence of different environment settings and to determine appropriate parameter settings for a comprehensive surface scan.

To decide which point cloud resolution to use for the configuration space analysis, we investigate the trade-off between point cloud resolution, coverage and run time, using an average male surface model [22]. In particular, we consider resolutions ranging from 0.5 m to 0.025 m. We use the kneedle algorithm [21] to find an optimal trade-off.

The environmental parameters studied are body geometry, couch height, and workspace constraints. First, we study the influence of different body geometries on the scanning coverage, namely half-cylinder, average male mesh, and surfaces extracted from CT scans. In particular, we use 10 different corpse surfaces extracted from CT scans acquired at the Institute of Legal Medicine at the University Medical Center Hamburg-Eppendorf (Legal Medicine, UKE). Please note, that we use a half-cylinder approach with quarter-sphere caps for path planning since the body geometry is usually not known before scanning. The half-cylinder model has a diameter of 0.6 m and a length of 1.60 m. For all body models, we neglect workspace constraints and use a surface point cloud resolution of 0.1 m with a couch height of 0.67 m.

In subsequent evaluations, we only use the CT scans as body models. Second, different couch heights are investigated, considering values of 0.67 m and 0.9 m, as both heights are within the range of CT table heights and autopsy table heights.

**Fig. 2:**
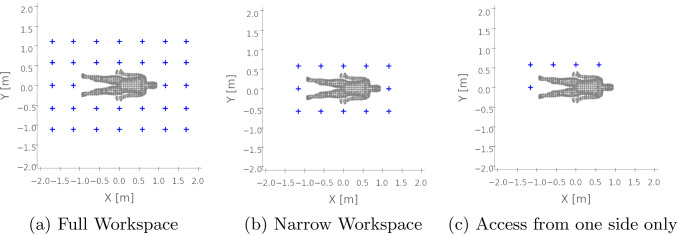
Workspace Constraints: different workspace constraints investigated in the experiments. Full workspace (left), narrow room (middle) and access from only one side (right)

Third, we evaluate the effect of workspace constraints visualized in Fig. 2. Confined environments are considered, as well as environments in which the body is only accessible from one side. Both environments correspond to typical constraints in the clinics.

For comparability, we always calculate the coverage for the corresponding high-resolution (0.01 m) point cloud and do not consider points from the back of the body, as they are hidden and therefore not accessible. A maximum of five different robot arm configurations per robot base position are considered to limit the scan time. We used the Python RoboticsToolbox [5] and the MoveIt! package [4] for the computational analysis. The code is executed on an Intel Core i7-13700K CPU with 64GB of RAM.

#### Real world evaluation

We evaluate the performance of our surface scanning approach in two real-world environments. More specifically, we use our robotic system in a laboratory setting without workspace constraints and in a forensic CT room with constraints similar to the evaluations in Fig. 2. In all scenarios, the robot is positioned randomly in the room. A maximum of three additional robot base positions per scan are used with a maximum of five robot arm configurations each.

**Fig. 3:**
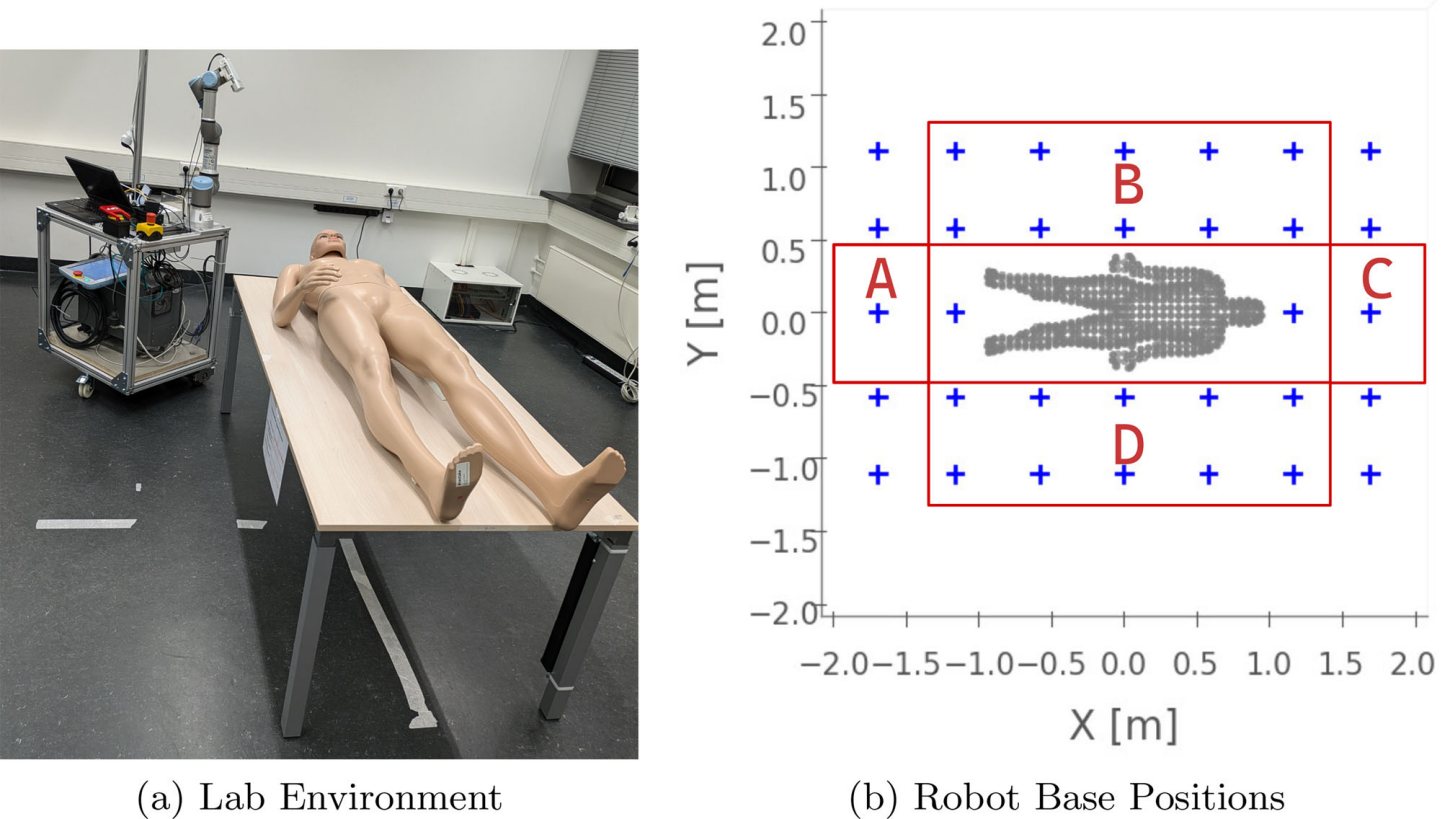
**Lab:** Lab environment (left) with autonomous scanning system and body phantom used for surface scanning. The investigated areas of starting positions for surface scanning are shown in (**b**)

In the laboratory setting (Fig. 3a), we use a male body phantom as scanning object. To serve as a reference, the surface geometry of the body phantom is acquired using a hand scanner (Artec Eva, Artec 3D, LU). Please note, that we used the Artec hand scanner as it provides a validated and well-established handheld scanning and processing pipeline. Within the scanning software (Artec Studio 17 Professional), several manual post-processing steps were performed, including manual fragment removal, fusion of multiple point clouds, and smoothed fusion.

To examine the repeatability of our system as a function of the initial robot base position in our laboratory setting, we divide the working region into four areas as defined in Fig. 3b. For each area, the robot base is randomly positioned and a surface scan is performed. This process is repeated ten times for each area, resulting in 40 surface scans with different starting positions. We evaluate our acquired surface scan using the coverage achieved compared to the reference scan from the hand scanner.

In addition to the experiments performed in a laboratory setting, we evaluated our system in a Legal Medicine environment. We scanned three male corpses and validated the acquired surface scan with the surface extracted from a corresponding CT scan (Incisive CT, Philips Healthcare, Netherlands). We used Slicer [7] to extract surfaces from the CT volume using the thresholding approach described by Neidhardt et al. [14]. Note that parts of the body were covered for addressing anonymity.

To determine the coverage achieved, we compare the acquired surface scan with the registered reference surface. We assume that a point is covered if a corresponding point in the reference scan is available and the registration error is less than twice the voxel size.

## Results

### Configuration space and path evaluation

The results of the trade-off analysis between point cloud resolution and runtime of the configuration space analysis are detailed in Table 1. An optimal solution is reached with a resolution of 0.1 m, yielding 96.43 % surface coverage and requiring 3.44 min for analysis of one robot base position.

**Table 1:** Configuration Space Analysis: Impact of the point cloud resolution used in the configuration space analysis on the coverage of the surface scan. Additionally, the corresponding mean time $t_\text {BP}$ for the configuration space analysis for a single robot base position in minutes is given

Resolution [*m*]	Surface Coverage [%]	$t_\text {BP}$ [min]
0.025	98.14	58.47
**0**.**1**	**96**.**43**	**3**.**44**
0.25	86.39	0.69
0.5	62.23	0.25

Bold indicates the resolution of the pointcloud which was finally used for the parameter study

**Table 2:** Configuration Space Analysis: Influence of different parameters on the achieved surface coverage (mean ± standard deviation) $[\%]$. The surface coverage for 1–4 different robot base positions is given

Number of Base Positions				
Parameter	1	2	3	4
Half-cylinder	70.91	95.81	99.60	100.00
Average male mesh	75.97	91.01	94.29	96.10
CT surface	$69.48 \pm 6.49$	$89.60 \pm 4.28$	$92.88 \pm 3.50$	$93.80 \pm 3.15$
Couch height (0.67 m)	$75.05 \pm 3.92$	$91.02 \pm 2.71$	$93.78 \pm 2.61$	$94.96 \pm 3.02$
Couch height (0.9 m)	$58.25 \pm 6.69$	$83.97 \pm 5.13$	$89.92 \pm 3.77$	$91.53 \pm 3.26$
Narrow room	$72.93 \pm 3.97$	$89.42 \pm 3.16$	$93.53 \pm 3.35$	$93.70 \pm 3.32$
One side only	$73.53 \pm 4.01$	$81.22 \pm 4.84$	$84.55 \pm 4.46$	$84.92 \pm 4.23$

**Table 3:** Lab: Surface coverage (mean ± standard deviation) [%] reached in the lab, depending on the different starting positions (A - D) and the number of robot base positions used. The coverage after the initial explorative scan is also given

Number of Base Positions				
Starting Area	Explorative Scan	1	2	3
A	$53.42 \pm 15.78$	$75.79 \pm 8.31$	$90.59 \pm 6.81$	$94.69 \pm 6.02$
B	$54.96 \pm 10.57$	$70.22 \pm 11.71$	$89.77 \pm 8.35$	$97.29 \pm 0.65$
C	$58.63 \pm 4.39$	$76.83 \pm 3.32$	$89.04 \pm 6.99$	$98.11 \pm 0.50$
D	$53.62 \pm 5.61$	$70.98 \pm 11.45$	$92.04 \pm 4.27$	$96.97 \pm 0.95$
Mean	$57.20 \pm 6.73$	$73.74 \pm 10.12$	$90.96 \pm 6.45$	$96.90 \pm 3.16$

The quantitative results of our configuration space analysis are shown in Table 2. Assuming a half-cylinder body geometry, full coverage is achieved using four robot base positions. Comparing the achieved coverage between three and four robot base positions, only a slight increase of 1 % is observed.

Using a half-cylinder body model for path planning, a coverage of 96.10 % was achieved for the average male mesh, while the different CT surfaces resulted in an average coverage of ${93.80 \pm 3.15}$ % (mean ± standard deviation).

Considering the detailed body geometry for path planning, similar results were obtained with a coverage of 96.43 % and ${94.96 \pm 3.02}$ % for average male mesh and CT surfaces, respectively.

Variations in couch height showed that increasing the height to 0.90 m resulted in a 3.43 % reduction in coverage. Workspace constraints limiting access to a single couch side reduced the potential surface coverage by 10 %, leaving coverage of 84.92 %. A narrow room results in similar coverage to no workspace constraints.

### Real world evaluation

**Fig. 4:**
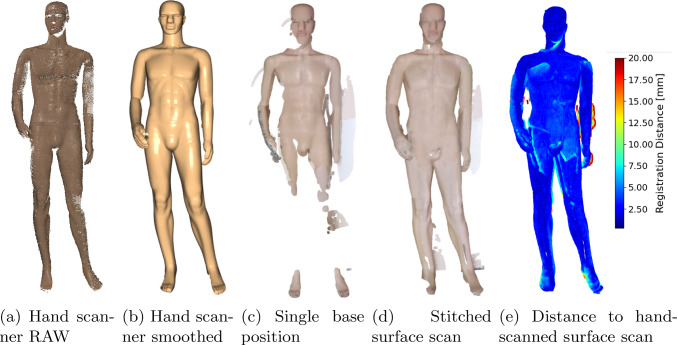
Example of an acquired surface scan after stitching. The reference surface acquired with the hand scanner (**b**) is shown as well as a surface scan using only one robot base position (**c**) and using all robot base positions (**d**). In **e** a color comparison regarding the absolute surface distance to the hand-scanned reference surface is shown

The results of our experiments performed in the lab are shown in Table 3. We reach a mean coverage of ${93.78 \pm 2.61}$ % with a mean difference of 6.01 mm compared to the hand-scanned reference surface. Similar coverage is achieved when evaluating the different starting positions (A-D). An exemplary surface scan is visualized in Fig. 4. The distance differences to the registered hand-scanned surface are indicated with color coding in Fig. 4e.

**Fig. 5:**
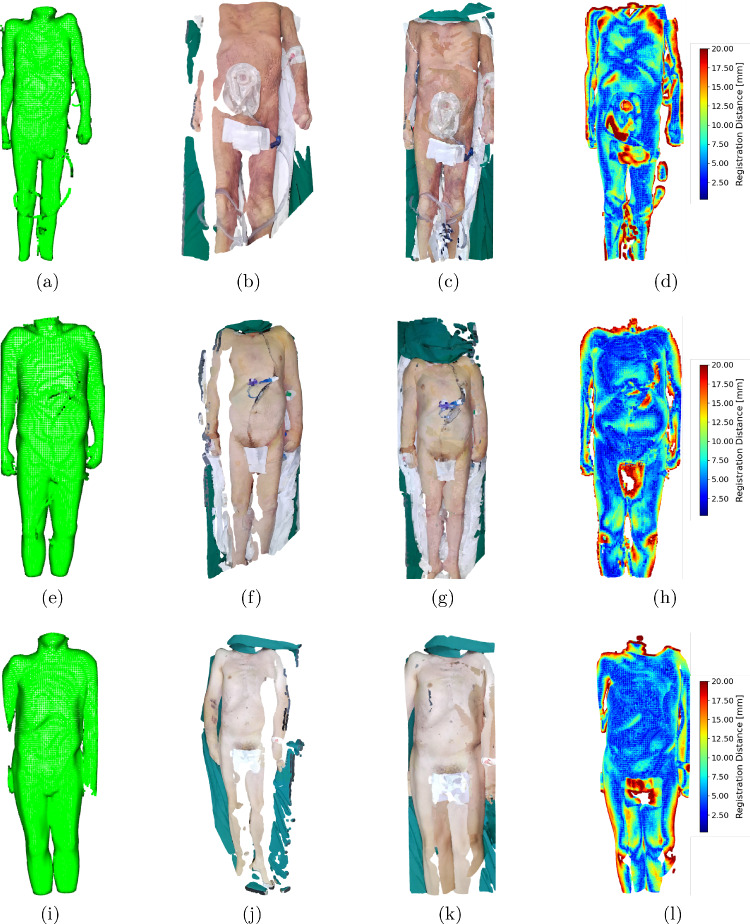
**Legal Medicine:** acquired surface scans for three different corpses. **a**,**e**,**i** show the surface extracted from the CT scan, **b**,**f**,**j** a surface scan from a single robot base position and **c**,**g**,**k** the surface scan using several robot base positions. In **d**,**h**,**l** a color comparison regarding the absolute surface distance to the registered CT surface is shown

The surface scans of three corpses acquired at Legal Medicine are shown in Fig. 5. Each row corresponds to a corpse and shows the extracted surface from the CT scan, an acquired surface scan from a single robot base position, the entire surface scan, and a color comparison regarding the absolute surface distance to the registered CT surface. The three surface scans reach a coverage of 90.80 %, 93.30 %, 93.26 % and a mean registration distance of 7.44 mm, 7.34 mm, 7.51 mm.

## Discussion

Our study demonstrates the capability of our mobile robot to perform autonomous surface scanning independent of body geometry and environmental settings.

### Configuration space and path evaluation

Our results demonstrate that using three robot base positions is generally sufficient to achieve good coverage across various experimental setups. For all body models, we achieve comparable results with a coverage of ${93.80 \pm 3.15}$ %, indicating the systems robustness to variations in body geometry. Note, that a coverage of 100 % would not be feasible because body parts in confined spaces, such as between the arms and legs and the torso, are impossible to access. Our results further indicate, that assuming a half-cylinder body model for path planning instead of detailed body geometries is reasonable for all body geometries studied. Therefore, we only need to perform a full configuration space analysis once with a half-cylinder body geometry approach, which enhances the real-time capability of our system.

No significant influence of the couch height on the scanning coverage is observed. However, it should be considered in the path planning process to avoid collisions between the robot and the corpse, as well as to ensure the use of the optimal depth range of the RGB-D camera.

The analysis of the workspace constraints shows that a narrow room still allows a scanning coverage of 90 %. However, if access is limited to one side of the couch, body geometries on the opposite side are particularly difficult to scan, resulting in a reduction in coverage to 84.92 %.

### Real world evaluation

While our configuration space analysis indicates an initial coverage of ${69.48 \pm 6.49}$ % for a single robot base position, the initial explorative scan from our lab experiments has a notably lower coverage of ${57.20 \pm 6.73}$ %. However, note, that the initial scan position is not optimized and a subsequent second scan from an optimized position yields a coverage of ${73.74 \pm 10.12}$ % that is even better than the expected coverage. This indicates that optimized positions are helpful to obtain good coverage. The need and importance of considering the environmental analysis from the initial scan into the subsequent path planning is demonstrated.

Using the optimized scanning path in the lab experiments, we achieved an overall coverage of ${96.90\pm 3.16}$% across all starting positions. This consistency demonstrates the robustness and flexibility of our scanning system and the independence of the initial position. On average, our mobile robot takes 16 min to complete a full-body scan in the lab at conservative speeds. Although this is slower than fixed CT-bound setups, such as the Virtoscan system [10], it approaches the performance of the Virtobot (15 min) and outperforms manual tripod scanning, which can take up to 30 min per side [6]. Future optimizations are expected to reduce the scan time.

Considering the qualitative analysis of the stitched point cloud in Fig. 4, point clouds obtained from a single robot base position show promising results. However, after stitching, overlapping areas between point clouds from multiple positions become slightly blurred, and distance differences to the reference scan appear mainly at the edges of the stitched sub-point clouds. In contrast, the hand-scanned reference scan appears smoother, due to extensive manual post-processing using the processing pipeline provided by the Artec software, also visible comparing Fig. 4a and Fig. 4b. We avoided such post-processing for the robotic scans in order to preserve the surface irregularities that are critical to our research. Therefore, the use of a more advanced and customized stitching algorithm, specifically focused on stitching partial point clouds [15], could improve the quality of our stitched surface scan. Our prototype uses the Azure Kinect, operating at distances between 0.5 and 1.5 m, with a depth resolution of 640 x 576 pixels. The resulting point density exceeds 10 points per cm$^2$, meeting the recommendations of Luchowski et al. for capturing key surface features [11]. The modular system design allows future integration of different imaging modalities, such as optical coherence scanning [23], to address specific forensic needs.

The surface scans acquired at Legal Medicine achieved a coverage of over 90 % compared to the CT surfaces, which indicates its potential for reliable corpse surface scanning. In Fig. 5j, a visible bruise on the arm illustrates the system’s ability to capture important legal medicine details. The surface scans show variability in lighting conditions, which could be addressed by adding external light sources to the system.

The extracted CT surface shows artifacts in its geometry, especially at the edges, due to the thresholding method used for extraction. This also explains the larger differences after the registration observed at the edges, where these artifacts are primarily concentrated.

**Fig. 6:**
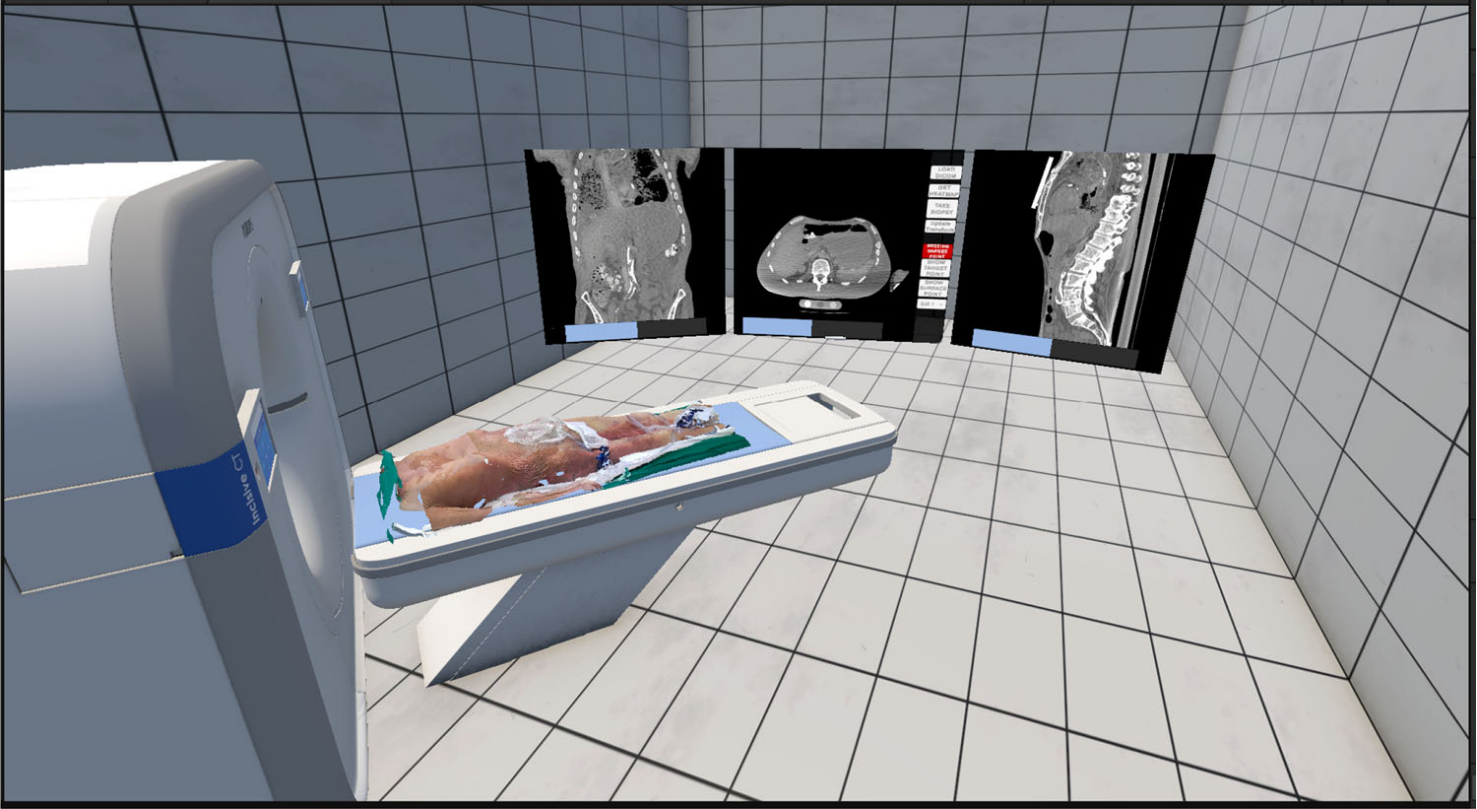
Possible visualization of the scanned surface in the Quest3

When applying our system in clinical practice, the acquired surface scans could be visualized in a mixed reality platform that combines different imaging modalities, such as the mixed reality platform from brainlab (Munich, GE). Figure 6 shows such a visualization in a Quest 3. The acquired surface scan is registered with the corresponding CT scan, allowing visualization of the CT slice corresponding to a particular point on the surface.

Future work on the system could focus on several key enhancements to improve precision and functionality. A more advanced stitching process for multiple point cloud stitching would lead to higher accuracy in spatial models. Optimizing the design of robot base is another priority; we aim to create a smaller base with better wheels to improve maneuverability. Moreover, automating the analysis of the surface scans would be another interesting focus for research, e.g., to detect and classify potential lesions or to measure their size [18, 30].

The presented robotic scanning system shows promising results in autonomously acquiring comprehensive surface scans in different environmental settings using three different robot base positions. Note, that the results and workspace considerations are also interesting for other similar robotic tasks, e.g., when using needles or instruments [13].

## Conclusion

In this study, we have presented a mobile and autonomous surface scanning robot designed to address the growing need for comprehensive and efficient legal medicine documentation. Our system provides an autonomous, cost-effective, and mobile alternative to traditional fixed or manual approaches. Our results show that the presented robotic scanning system is a promising approach to enable autonomous surface scanning in a variety of environments. The conducted analysis underlines the importance of considering environmental settings for calculating the optimal scanning path. Future work will focus on optimizing the system design to further improve robustness and automation, as well as improving the surface capture device and subsequent stitching process.

## Supplementary Information

Below is the link to the electronic supplementary material.

Supplementary file 1 (mp4 10699 KB)

## References

[1] Bruyninckx H (2001) Open robot control software: the orocos project. In: 2001 IEEE Int Conf Robot Autom, pp 2523–2528, 10.1109/ROBOT.2001.933002

[2] Buck U, Buße K, Campana L, Schyma C (2018) Validation and evaluation of measuring methods for the 3d documentation of external injuries in the field of forensic medicine. Int J Legal Med 132(2):551–561. 10.1007/s00414-017-1756-6 10.1007/s00414-017-1756-6 29260394

[3] Campana L, Breitbeck R, Bauer-Kreuz R, Buck U (2016) 3d documentation and visualization of external injury findings by integration of simple photography in ct/mri data sets (iproject). Int J Legal Med 130(3):787–797. 10.1007/s00414-015-1274-3 26496803 10.1007/s00414-015-1274-3

[4] Coleman D, Sucan I, Chitta S, Correll N (2014) Reducing the barrier to entry of complex robotic software: a moveit! case study. arXiv 10.6092/JOSER_2014_05_01_P3

[5] Corke P, Haviland J (2021) Not your grandmother’s toolbox–the robotics toolbox reinvented for python. In: 2021 IEEE Int Conf Robot Autom, pp 11357–11310.1109/ICRA48506.2021.9561366

[6] Ebert LC, Ptacek W, Breitbeck R, Fürst M, Kronreif G, Martinez RM, Thali M, Flach PM (2014) Virtobot 2.0: the future of automated surface documentation and ct-guided needle placement in forensic medicine. Forensic Sci Med Pathol 10(2):179–186. 10.1007/s12024-013-9520-9 24474435 10.1007/s12024-013-9520-9

[7] Fedorov A, Beichel R, Kalpathy-Cramer J, Finet J, Fillion-Robin JC, Pujol S, Bauer C, Jennings D, Fennessy F, Sonka M et al (2012) 3d slicer as an image computing platform for the quantitative imaging network. Magn Reson Imaging 30(9):1323–1341. 10.1016/j.mri.2012.05.001 22770690 10.1016/j.mri.2012.05.001 PMC3466397

[8] Fischler MA, Bolles RC (1981) Random sample consensus: a paradigm for model fitting with applications to image analysis and automated cartography. Commun ACM 24(6):381–395. 10.1145/358669.358692

[9] Hart P, Nilsson N, Raphael B (1968) A formal basis for the heuristic determination of minimum cost paths. IEEE Trans Syst Sci Cybern 4(2):100–107. 10.1109/TSSC.1968.300136

[10] Kottner S, Ebert LC, Ampanozi G, Braun M, Thali MJ, Gascho D (2017) Virtoscan - a mobile, low-cost photogrammetry setup for fast post-mortem 3d full-body documentations in x-ray computed tomography and autopsy suites. Forensic Sci Med Pathol 13(1):34–43. 10.1007/s12024-016-9837-2 28144846 10.1007/s12024-016-9837-2

[11] Luchowski L, Pojda D, Tomaka AA, Skabek K, Kowalski P (2021) Multimodal imagery in forensic incident scene documentation. Sensors 21(4):1407. 10.3390/s21041407 33671419 10.3390/s21041407 PMC7922848

[12] Nawrat Z, Krawczyk D (2023) Robot-Based Medicine. Robots in Medicine: Mobile Robots Versus Mobile Decision, Necessity Versus Possibility and Future Challenges. In: Azar AT, Kasim Ibraheem I, Jaleel Humaidi A (eds) Mobile Robot: Motion Control and Path Planning, Studies in Computational Intelligence, vol 1090. Springer International Publishing, Cham, p 127–162, 10.1007/978-3-031-26564-8_5

[13] Neidhardt M, Gerlach S, Mieling R, Laves MH, Weiß T, Gromniak M, Fitzek A, Möbius D, Kniep I, Ron A et al (2022) Robotic tissue sampling for safe post-mortem biopsy in infectious corpses. IEEE Trans Med Robot Bio 4(1):94–105 10.1109/TMRB.2022.3146440 PMC8956373 35582701

[14] Neidhardt M, Gerlach S, Mieling R, Laves MH, Weiß T, Gromniak M, Fitzek A, Möbius D, Kniep I, Ron A, Schädler J, Heinemann A, Püschel K, Ondruschka B, Schlaefer A (2022) Robotic tissue sampling for safe post-mortem biopsy in infectious corpses. IEEE Trans Med Robot Bionics 4(1):94–105. 10.1109/TMRB.2022.3146440 35582701 10.1109/TMRB.2022.3146440 PMC8956373

[15] Oğuz E, Doğan Y, Güdükbay U, Karaşan O, Pınar M (2024) Point cloud registration with quantile assignment. Mach Vis Appl. 10.1007/s00138-024-01517-3

[16] Pan J, Chitta S, Manocha D (2012) Fcl: A general purpose library for collision and proximity queries. In: 2012 IEEE Int Conf Robot Autom, pp 3859–3866, 10.1109/ICRA.2012.6225337

[17] Park F, Martin B (1994) Robot sensor calibration: solving ax=xb on the euclidean group. IEEE Trans Robot 10(5):717–721. 10.1109/70.326576

[18] Patel Y, Shah T, Dhar MK, Zhang T, Niezgoda J, Gopalakrishnan S, Yu Z (2024) Integrated image and location analysis for wound classification: a deep learning approach. Sci Rep 14(1):7043. 10.1038/s41598-024-56626-w 38528003 10.1038/s41598-024-56626-w PMC10963767

[19] Urbanová P, Hejna P, Jurda M (2015) Testing photogrammetry-based techniques for three-dimensional surface documentation in forensic pathology. Forensic Sci Int 250:77–86. 10.1016/j.forsciint.2015.03.005 25818581 10.1016/j.forsciint.2015.03.005

[20] Rusinkiewicz S, Levoy M (2001) Efficient variants of the icp algorithm. In: Proceedings Third International Conference on 3-D Digital Imaging and Modeling, pp 145–152, 10.1109/IM.2001.924423

[21] Satopaa VA, Albrecht JR, Irwin DE, Raghavan B (2011) Finding a "kneedle" in a haystack: Detecting knee points in system behavior. 2011 31st International Conference on Distributed Computing Systems Workshops pp 166–171. 10.1109/ICDCSW.2011.20

[22] Schlehlein H, II BWH, Quardokus EM, Bueckle A, Börner K (2022) Hubmap ccf 3d reference object library. https://hubmapconsortium.github.io/ccf/pages/ccf-3d-reference-library.html, version 1.3, accessed December 15, 2022

[23] Sprenger J, Neidhardt M, Latus S, Grube S, Fischer M, Schlaefer A (2022) Surface scanning for navigation using high-speed optical coherence tomography. Curr Dir Biomed Eng 8(1):62–65. 10.1515/cdbme-2022-0016

[24] Stanford Artificial Intelligence Laboratory et al. (2018) Robotic operating system. https://www.ros.org, online; accessed 2024-12-01

[25] Sucan IA, Moll M, Kavraki LE (2012) The open motion planning library. IEEE Robot Autom Mag 19(4):72–82. 10.1109/MRA.2012.2205651

[26] Thali MJ, Braun M, Buck U, Aghayev E, Jackowski C, Vock P, Sonnenschein M, Dirnhofer R (2005) VIRTOPSY—Scientific Documentation, Reconstruction and Animation in Forensic: Individual and Real 3D Data Based Geo-Metric Approach Including Optical Body/Object Surface and Radiological CT/MRI Scanning. J Forensic Sci 50(2):JFS2004290–15. 10.1520/JFS2004290 15813556

[27] Sieberth T, Meindl M, Sagmeister B, Franckenberg S, Ptacek W (2024) Cost-effective 3d documentation device in forensic medicine. Forensic Sci Int 357:112005. 10.1016/j.forsciint.2024.112005 38522324 10.1016/j.forsciint.2024.112005

[28] Universal Robots and FZI Research Center for Information Technology (2025) Universal Robots ROS Driver. https://github.com/UniversalRobots/Universal_Robots_ROS_Driver, accessed: 2025-06-04

[29] Buck U, Naether S, Räss B, Jackowski C, Thali MJ (2013) Accident or homicide - virtual crime scene reconstruction using 3d methods. Forensic Sci Int 225(1):75–84. 10.1016/j.forsciint.2012.05.015 22727689 10.1016/j.forsciint.2012.05.015

[30] Zhou J, Zhou Z, Chen X, Shi F, Xia W (2023) A deep learning-based automatic tool for measuring the lengths of linear scars: forensic applications. Forensic Sci Res 8(1):41–49. 10.1093/fsr/owad010 37415798 10.1093/fsr/owad010 PMC10265961

[31] Zhou QY, Park J, Koltun V (2018) Open3d: A modern library for 3d data processing. arXiv:1801.09847

